# Functional Equivalence of Imagined vs. Real Performance of an Inhibitory Task: An EEG/ERP Study

**DOI:** 10.3389/fnhum.2016.00467

**Published:** 2016-09-16

**Authors:** Santiago Galdo-Alvarez, Fidel M. Bonilla, Alberto J. González-Villar, María T. Carrillo-de-la-Peña

**Affiliations:** ^1^Department of Clinical Psychology and Psychobiology, Universidade de Santiago de CompostelaSantiago de Compostela, Spain; ^2^Laboratory of Experimental Psychology, Faculty of Psychology, University El BosqueBogotá, Colombia

**Keywords:** functional equivalence, inhibition, stop-signal task, motor imagery, ERPs, time-frequency EEG analyses

## Abstract

Early neuroimaging and electrophysiological studies suggested that motor imagery recruited a different network than motor execution. However, several studies have provided evidence for the involvement of the same circuits in motor imagery tasks, in the absence of overt responses. The present study aimed to test whether imagined performance of a stop-signal task produces a similar pattern of motor-related EEG activity than that observed during real performance. To this end, mu and beta event-related desynchronization (ERD) and the Lateralized Readiness Potential (LRP) were analyzed. The study also aimed to clarify the functional significance of the Stop-N2 and Stop-P3 event-related potential (ERPs) components, which were also obtained during both real and imagined performance. The results showed a common pattern of brain electrical activity, and with a similar time course, during covert performance and overt execution of the stop-signal task: presence of LRP and Stop-P3 in the imagined condition and identical LRP onset, and similar mu and beta ERD temporal windows for both conditions. These findings suggest that a similar inhibitory network may be activated during both overt and covert execution of the task. Therefore, motor imagery may be useful to improve inhibitory skills and to develop new communicating systems for Brain-Computer Interface (BCI) devices based on inhibitory signals.

## Introduction

During the last decades, Brain-Computer Interface (BCI) communicating systems are being developed successfully for a variety of clinical (Mak and Wolpaw, 2009) and non-clinical (Blankertz et al., 2012) applications. These systems are based mostly on the assumption that the mental rehearsal of an action recruits the same neural mechanisms as its real performance. In particular, the simulation theory, also known as the functional equivalence hypothesis (Jeannerod, 2001), suggests that a similar cortical network, including primary areas, is involved during both mental practice of a movement and its overt execution.

The assumption of a functional equivalence challenges the classical hierarchical view of the motor system. Since Penfield and colleagues reported that stimulation of specific neurons in the primary motor cortex (M1) resulted in movements following a somatotopic representation (Penfield and Boldrey, 1937; Penfield and Rasmussen, 1950), it has been generally assumed that M1 plays the role of a pure executor receiving orders from superior motor centers. In support of this view, former neuroimaging studies on motor imagery confirmed that primary and secondary motor areas were recruited during motor execution, but only secondary areas showed activation during mental practice of the same movements (Roland et al., 1980; Decety et al., 1988). Thus, they concluded that M1 is not activated when motor output is absent.

However, since then, many studies have questioned the hierarchical assumption and provided support for the functional equivalence hypothesis. Thus, various fMRI studies reported that the same network, including M1, was activated in motor imagery (Ersland et al., 1996; Porro et al., 1996; Roth et al., 1996; Lotze et al., 1999; Gerardin et al., 2000; Stippich et al., 2002). In several of these studies, it became clear that this activation could not be explained by subtle motor activity, as trials showing any EMG activity were discarded (Lotze et al., 1999; Gerardin et al., 2000; Lafleur et al., 2002).

Additional support for this hypothesis stems from event-related potential (ERPs) studies using the motor imagery paradigm (Galdo-Alvarez and Carrillo-de-la-Peña, 2004; Carrillo-de-la-Peña et al., 2006, 2008; Kranczioch et al., 2009; Hohlefeld et al., 2011). Although the EEG/ERP technique is characterized by a low spatial resolution, it provides a direct online measure of cortical activation and allows testing whether similar processes are taking place in the same temporal interval (Cohen, 2014; Luck, 2014). Several studies have claimed that one particular ERP component, the lateralized readiness potential (LRP), is generated in M1. The LRP is obtained from central electrodes and reflects the lateralized portion of motor ERPs. The main evidence for M1 as the source of this component is the inversion of polarity found for lower limb movements, as compared to hand movements. Brunia (1980) explained the inversion by the somatotopical distribution of the neurons on the M1: hands are represented in the lateral surface of precentral gyrus, whereas legs are represented in the medial surface. In addition, source reconstruction of LRP activity using EEG (Böcker et al., 1994a,b) and MEG (Praamstra et al., 1999) dipole modeling is consistent with the activation of M1.

Galdo-Alvarez and Carrillo-de-la-Peña (2004) reported that the LRP was present, although with a smaller amplitude, during covert performance, a result that the authors interpreted as evidence for the activation of M1 during motor imagery. Further research (Carrillo-de-la-Peña et al., 2006, 2008) confirmed this finding and provided evidence of functional equivalence of overt and covert actions; e.g., similar timing for simple and sequential or complex movements, inversion of polarity for lower limbs, and similar activation for hand selection. In fact, Hohlefeld et al. (2011) reported that overt and covert movements differed in stimulus processing at early stages of response selection, rather than in motor processing.

From a different perspective, several studies have explored how motor imagery affects EEG oscillations related to movement, i.e., mu and beta bands recorded over the somatosensory and motor areas. Consistent with this, a similar motor-related EEG pattern generally referred to as mu and beta event-related desynchronization (ERD) has been found during motor imagery and actual movement (Pfurtscheller et al., 2006; Stavrinou et al., 2007; Nam et al., 2011). The findings of numerous studies using Transcranial Magnetic Stimulation (TMS) also indicate that motor imagery significantly increases corticospinal excitability (Mizuguchi et al., 2009; Roosink and Zijdewind, 2010; Williams et al., 2012).

Overall, the data on ERPs, EEG dynamics and TMS during motor imagery provide support for the functional equivalence hypothesis. However, the above-mentioned studies analyzed selection, preparation or execution of simple motor responses. In natural situations, motor skills and actions require fine executive processing that involves coding strength, direction and other muscle parameters and also the ability to reset and inhibit ongoing performance. It would therefore be interesting to explore the brain electrical activity during the covert performance of inhibitory tasks.

The go/no-go and the stop-signal tasks are the paradigms most commonly used to study response inhibition, understood as the ability to suppress, withhold, delay or interrupt ongoing or planned actions. The stop-signal task explores inhibition of an already initiated response, i.e., action cancellation, and thus implies greater inhibitory pressure on response-related processes than the go/no-go paradigm (Swick et al., 2011). Two fronto-central ERP components have been associated with performance of the stop-signal task: Stop-N2, a possible index of the conflict between an initiated go response and the stop signal, and Stop-P3, a component whose interpretation is still open to debate. The P3 amplitude is larger in successful than unsuccessful stop (US) trials and in subjects with fast stop performances (requiring greater inhibitory activation; Dimoska et al., 2006), supporting its interpretation as an index of inhibitory efficiency. It has been suggested that the source of Stop-P3 may be in the premotor cortex, a region believed to be responsible for mediating stop-signal inhibition (Kok et al., 2004; Ramautar et al., 2006). Nevertheless, its latency appears to be too late to reflect the initial process of voluntary response inhibition, and it has thus been interpreted as an index of evaluation of the inhibitory process (Huster et al., 2013). It has been also suggested that in no-go and stop trials this positivity may be modulated by the lack of negative activity associated with motor preparation (Kok, 1986; Verleger et al., 2006).

Although the recording of brain activity during the covert performance of an inhibitory task could provide additional support for the functional equivalence hypothesis, as far as we know, there is only one study comparing actual and imagined performance of a stop-signal task (González-Villar et al., 2016). Using auditory stimuli as stop signals, they found similar Stop-N2, Stop-P3 and mu and beta ERD in mental essays and real performance of the task, but did not study the LRP as a possible index of M1 activation.

Thus, the main aim of the present study was to test whether covert performance of a stop-signal task produces the same pattern of motor-related EEG activity observed during real performance. To this end, mu and beta ERD and the LRP were obtained during both imagined and real performance of go and stop trials. A similar pattern on these indices during both conditions may support the general applicability of the functional equivalence hypothesis to tasks that exert increased executive control over motor performance, as the stop-signal task does.

An additional objective was to replicate the previous study, testing whether the ERP indices that characterize response cancellation (i.e., Stop-N2 and Stop-P3) are also present during the covert performance of the Stop-signal task, using visual stimuli both as targets and as stop signals. Specifically, the presence of Stop-P3 in the covert condition could provide indirect evidence on the activation of an inhibitory network during imagery.

The present study also attempted to clarify the functional meaning of Stop-N2 and Stop-P3. Comparison of ERP components (LRP, Stop-N2 and Stop-P3) produced in US, successful stop (SS) and Imagined Stop (IS) trials may shed some light on the role of motor execution or outcome correction processes in classical ERP inhibition indices.

## Materials and Methods

### Sample

A total of 18 students (5M, 13F) ranging from 19 to 32 years (mean = 20.89; SD = 1.72) participated voluntarily in the study. All were right-handed, according to the Edinburgh handedness inventory, and reported normal or corrected vision. None of them presented a history of neurological or psychiatric disorders, or drug abuse. Informed consent was received from all the participants, in accordance with the Declaration of Helsinki.

### Stimuli and Apparatus

The primary task consisted of a choice reaction task in response to white arrows pointing to the left or the right side (stimulus duration: 500 ms; mean interval between stimulus onsets: 2100 ms), which indicated the hand that participants had to respond with. The start of each trial was indicated by the appearance of a fixation cross in the center of the screen. Then, the white arrows substituted the fixation cross. The arrow consisted of an arrowhead and a tail and had a size of 2.1° · 1.4° of visual angle. In 30% of trials, a red arrow (stop signal) indicated that subjects had to cancel the already prepared response.

The task was designed and presented using the STIM program (Neuroscan Labs). The stimuli were presented on a 15″ screen located at a distance of 100 cm from the subjects. Participants responded using a response box held in their hands.

### Design and Procedure

Participants were seated comfortably in an armchair in a dimly lit, sound attenuated room. They were instructed to look at the fixation cross in the center of the screen and to press a button with their right or left thumb according to the direction indicated by the white arrow. They were informed that in some trials a red arrow might appear after the white arrow, indicating that the response should be canceled. Subjects were instructed to respond as quickly as possible to the white arrow and not to wait for the appearance of the stop signal. They completed some practice trials before the first block of experimental trials.

In the real condition, the time interval between the onset of go signals and stop signals was 300 ms in the first trial and was then changed according to the subject’s performance (ranging from 160 to 400 ms in 40 ms steps). The interval was altered using the staircase-tracking algorithm that adjusts the go-stop interval in a certain trial depending on the results of the previous stop trial (Band and van Boxtel, 1999). This algorithm produces a distribution around $\frac{1}{2}$ of successful and $\frac{1}{2}$ of unsuccessful response-inhibited trials. If the response in the previous stop trial was correctly inhibited, the interval between go and stop signals in the next stop trial was 40 ms longer, also increasing the difficulty of successful inhibition; if the subject responded in the previous stop trial, the interval between signals in the next stop trial was 40 ms shorter, in order to facilitate inhibition (Logan and Cowan, 1984).

In the imagined condition, subjects were instructed to imagine as vividly as possible responding with the hand of the side pointed by the white arrow, and to withhold the response (like braking suddenly) when the stop signal appeared. They had to keep their hands on the response box, as in real performance. In this condition, due to the lack of response feedback, the Go-Stop signal interval was fixed at 300 ms.

The task for each condition consisted of 280 trials, 70% of them were Go (196 trials, 98 for each direction) and 30% Stop (84 trials, 42 for each direction). The order of the tasks was always the same: first, overt execution and then covert performance. This procedure was used to ensure more effective mental rehearsal after real practice, as revealed by previous studies (Cunnington et al., 1996; Carrillo-de-la-Peña et al., 2006). Participants were allowed a 5 min rest between both tasks.

### Psychophysiological Recording and Data Analyses

The EEG was recorded from 28 electrode sites (10–20 international system) referenced to the left and right mastoids, using pure tin electrodes attached to a fabric cap (Electro-Cap International, Inc., Eaton, OH, USA). The electrooculogram (EOG) was recorded from sites above and below the left eye and from electrodes lateral to each eye. The AFz electrode served as ground electrode. Electrode impedances were kept below 10 kΩ. The EEG signals were digitized online with Neuroscan equipment (Neuroscan Laboratories, version 4.1), amplified 10,000 times (SynAmp Model 5083 amplifier), filtered using a band-pass between 0.1 and 100 Hz and a notch filter of 50 Hz, and sampled at a rate of 500 Hz.

The EEG data were analyzed using the EEGlab 12.02 toolbox (Delorme and Makeig, 2004). The data were resampled to 250 Hz and re-referenced to an average-reference. Poorly recorded channels were replaced by spherical-spline interpolation and EEG segments containing large ocular or other artifacts were rejected after visual inspection. The data were digitally filtered using a low-pass 30 Hz FIR filter. An Independent Component Analysis algorithm was used to remove components associated with ocular artifacts. The EEG data used for the ERP analyses were baseline corrected from −200 to 0 ms. Epochs were extracted from 200 ms pre-stimulus to 900 ms post-stimulus, and were extracted time-locked to go stimuli (white arrows) and to the stop stimuli (red arrows; only for N2 and P3 analyses). The ERPs used to measure the N2 wave were filtered with a 2–12 Hz band-pass filter to avoid overlap with other ERP waves.

The stop-signal task is complicated by the fact that the activity to the stop stimuli overlaps with the activity evoked by the previous go signal. To resolve this, we subtracted the activity evoked by go trials from the ERPs obtained in stop trials. First, we calculated the percentage of SS and US trials for each subject, and this percentage was used to select go trials in the following way: if the participant had a 45% of US in all stop trials, the 45% of the fastest go epochs were used as the pool of trials to make the subtraction of the US minus Fast Go trials. The remaining 55% of the slowest go trials were used as the pool to make the subtraction SS minus Slow Go trials. A random go epoch (selected from its respective pool of Go epochs) was then assigned to each stop epoch. Finally, stop and go epochs were aligned by the go signal, and the subtraction was computed. This method was applied in previous studies (Kok et al., 2004; Ramautar et al., 2006).

The LRP was obtained by the average method proposed by Coles (1989), i.e., it was computed by subtracting ERP activity at C3 minus C4 for the right responses and C4 minus C3 for the left responses, and then averaging the resulting difference waveforms. This removes non-motor contribution from this index of lateralized activity associated with response preparation. LRPs were obtained for each trial (go, stop) and task (overt, covert). Also, the topographical distributions of LRPs were calculated using the method described by Praamstra and Seiss (2005), applying the average method to obtain LRP from each pair of contralateral electrodes (e.g., F3/F4, FC3/FC4…; only for go trials in both tasks).

Mean amplitudes were obtained for N2 (200–260 ms interval) and P3 (260–450 ms interval) at the FCz electrode site. As different numbers of trials were presented for the different conditions, mean amplitudes were measured instead of peak amplitudes to prevent confusion due to different signal-to-noise-ratios.

Time-Frequency Analysis was performed by convolving the EEG data with a family of complex Morlet wavelets ranging in frequency from 3 to 30 Hz in 27 linearly increasing steps, and with logarithmically increasing cycles, from three cycles at the lowest frequency to eight at the highest frequency. Power data obtained after convolution was baseline corrected by transforming the power change of each time-frequency pixel to dB, relative to the mean power in the baseline interval (−400 to −100 ms) of each frequency.

As the frequencies of interest here are more prominent around Rolandic areas, we first averaged spectrograms of C3 and C4 electrodes. For analysis of mu and beta oscillations, time-frequency windows were selected after averaging the spectrograms for Trial (go, stop) and Task (overt, covert) together, to avoid making assumptions about condition differences. We observed that mu band had two peaks at different latencies (at around 450 and 700 ms, respectively), and we therefore extracted two different windows (from 300 to 550 ms and from 600 to 900 ms) in the 9–13 Hz range. For the beta band, we extracted the mean power from 200 to 550 ms between 18 and 24 Hz.

### Statistical Analysis

Behavioral and ERP parameters were analyzed by considering the available measures in the different conditions. Thus, given the lack of motor response in motor imagery conditions, we carried out *t* tests to examine differences in behavioral performance reaction times (RTs) between the overt go response and overt US trials.

In order to assess the possible existence of LRPs during covert motor performance, we carried out one-sample Wilcoxon tests for the mean of five consecutive windows of 50 ms each, with a step size of 10 ms between windows (i.e., each window had an overlap of 40 ms with the prior window), starting 40 ms before the peak latency (approximately 370 ms). If significant differences were found for all the windows, we could conclude that the waveforms deviated significantly from baseline and thus that LRPs were also present during mental rehearsal of movements in the different conditions of the task.

LRP mean amplitudes were measured in the 300–400 interval. The LRP onset latencies were determined using the jackknife procedure. Therefore, 18 different grand averages for each of the experimental conditions were computed by omitting one of the participants from each grand average. The onset was subsequently measured using the method proposed by Schwarzenau et al. (1998), which assumes that the onset of correct preparation corresponds to the intersection point of two straight lines, one fitted to the baseline and another to the rising slope of the LRP.

For the LRP, N2 and P3 mean amplitudes and the beta and mu ERD power, repeated-measures analysis of variances (ANOVAs) were carried out with two within-subject factors (Trial: go, stop; Task: overt, covert). In these analyses, overt response stop trials included only those trials in which successful inhibition was observed. Possible differences between tasks in go LRP topography were analyzed using a repeated measures ANOVA on LRP mean amplitudes (200–400 ms), with task (overt, covert), and electrode pair (F3/F4, FC3/FC4, C3/C4, CP3/CP4, P3/P4) as within-subject factors. The LRP onsets were subsequently analyzed by means of repeated-measures ANOVA with two within-subject factors (Trial: go, stop; Task: overt, covert). The *F* values in the latter case were corrected using the formula *F* = F/(*n* − 1)^2^, as recommended when performing the jackknife procedure for statistical analyses (Ulrich and Miller, 2001).

To clarify the effect of successful vs. unsuccessful performance of the stop-signal task, additional repeated-measures ANOVAs were carried out with the within-subject factor Performance (SS, US, IS) for the same parameters.

## Results

### Behavioral Performance

Table 1 shows behavioral indices for go and stop trials (as means of left and right hand responses). For go trials, the data included percentages of hits, errors and missing responses, as well as RTs for hits and errors. For stop trials, the percentage of US trials and their RTs, as well as mean stop signal delay (SSD) values and stop signal reaction times (SSRTs) are provided^1^.

**Table 1:** **Behavioral parameters for the overt performance of the stop-signal task**.

**Go**
% Hits	93.4 (7.5)
% Errors	2.5 (3.2)
% Missing	3.7 (6.6)
RTs for hits	453 (94)
RTs for errors	342 (109)
**STOP**
% US	50 (17)
US RTs	396 (60)
SSD	250 (47)
SSRT	203 (60)

*RTs, reaction times; US, unsuccessful stop trials; SSD, stop signal delay; SSRT, stop signal reaction time*.

The percentage of US was about 50%, as expected given the use of the staircase tracking algorithm. RTs were faster in US trials than in go trials (*t* = 5.8, *p* < 0.001).

### LRP

**Figure 1:**
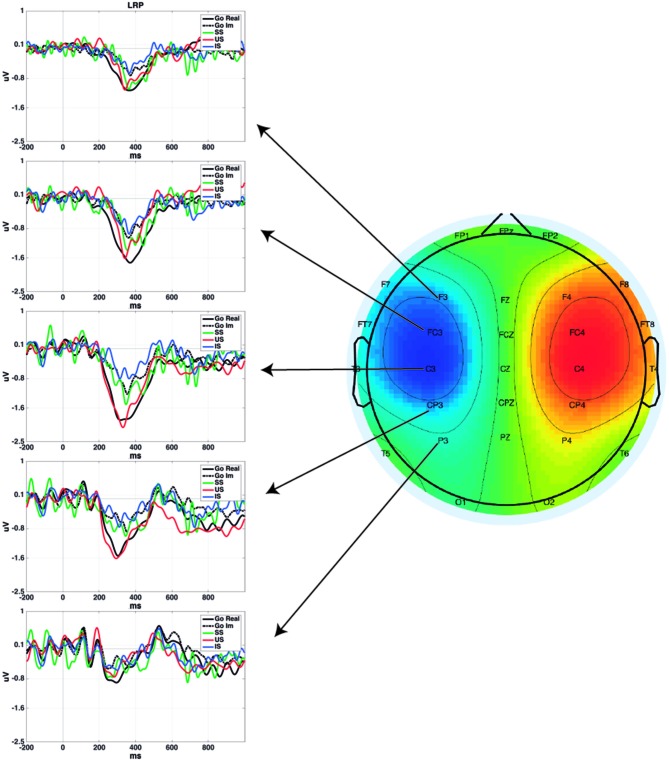
**Lateralized Readiness Potential (LRP) time-locked to the go signal for each condition in different scalp locations.** Plotted grand averages of Successful Stop (SS) and Unsuccessful Stop (US) were computed using 12 participants, while Go Real, Go Im and Imagined Stop (IS) were computed using 18 participants. Topography represents the mean LRP amplitude of all conditions from 200 to 400 ms.

**Figure 2:**
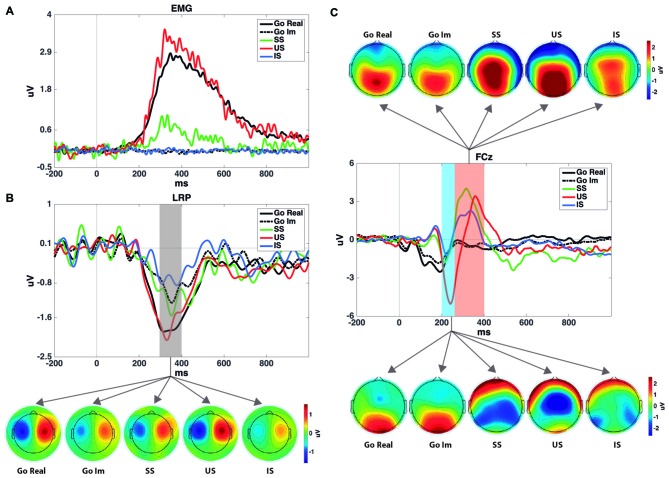
**(A)** Rectified electromyogram (EMG) for each condition. It shows that no EMG activity was registered after stimulus presentation during the imagined task. **(B)** LRP time-locked to the go signal and the topographies of the shaded area. SS and US grand averages of the LRPs were computed using 12 participants, while Go Real, Go Im and IS were computed using 18 participants. Topographies were calculated using the method described by Praamstra and Seiss (2005). **(C)** Event-related potential (ERPs) for each task and condition at the FCz electrode site and their topographies in the windows selected to measure N2 and P3 components. Note that go trials were averaged time-locked to the go signal, while SS, US and IS were averaged time-locked to the stop signal and with go-stimulus ERPs subtracted.

Figure 1 presents the LRP obtained in different pairs of electrode sites and the scalp distribution of the component. Figure 2 presents the average waveforms of EMG, LRP and stimulus-locked components (N2, P3) obtained from go and stop trials in both overt and covert performance, as well as the scalp distribution for each component.

One-sample Wilcoxon tests were performed to confirm the existence of LRPs in covert response trials. All comparisons revealed significant differences from 0, and therefore we can conclude that the LRP is present in motor imagery for both go and stop trials (Table 2). The mean values and standard deviations for all the ERP parameters measured, including LRP, are shown in Table 3.

**Table 2:** **One-sample Wilcoxon tests for covert trials Lateralized Readiness Potential (LRP) amplitude**.

Condition	Interval	Voltage average (microvolts)	Wilcoxon value
Go	340–390	−0.95	−3.7***
	350–400	−1.01	−3.6***
	360–410	−0.87	−3.4***
	370–420	−0.88	−3.3***
	380–430	−0.84	−3.2**
Stop	340–390	−0.34	−2.0*
	350–400	−0.34	−2.3*
	360–410	−0.46	−2.1*
	370–420	−0.40	−2.1*
	380–430	−0.26	−2.1*

*p < 0.05 **p < 0.01 ***p < 0.001.

**Table 3:** **Mean and standard deviations (in parentheses) for the measured event-related potential (ERP) parameters and mu and beta eventrelated desynchronization (ERD)**.

		LRP Onset (ms)	LRP Amp. (μV)	N2 Amp. (μV)	P3 Amp. (μV)	beta ERD 200–550 (dB)	mu ERD 300–550 (dB)	mu ERD 600–900 (dB)
Overt performance	Go	176 (3)	−1.7 (1.2)	−1.3 (1.8)	−0.5 (1.4)	−1.6 (1.1)	−2.6 (1.5)	−2.2 (1.5)
	Successful stop	204 (21)	−0.9 (0.8)	−0.8 (2.2)	2.0 (3.0)	−1.9 (1.2)	−2.6 (1.7)	−2.9 (1.5)
	Unsuccessful stop	182 (6)	−1.6 (1.3)	−3.7 (4.4)	1.1 (2.9)	−1.7 (1.2)	−2.4 (1.7)	−3.4 (2.2)
Covert performance	Go	196 (8)	−0.9 (0.9)	−1.1 (1.6)	−0.4 (0.9)	−0.7 (0.8)	−1.6 (1.2)	−1.0 (1.0)
	Stop	222 (35)	−0.6 (0.9)	−0.4 (2.0)	1.0 (1.6)	−0.8 (0.7)	−1.6 (1.4)	−1.2 (2.0)

*Note: LRP for Unsuccessful Stop (US) data were obtained from 12 participants; for the other parameters, EEG recordings from the 18 participants were used*.

The repeated-measures ANOVA (Trial × Task) for LRP amplitude showed significant main effects of Trial (*F*_(1,17)_ = 22.4; *p* < 0.001) and Task (*F*_(1,17)_ = 9.3; *p* = 0.007), but no interaction effect (*F*_(1,17)_ = 3.0; *p* = 0.1). The LRP amplitude was larger in go than in stop trials, and it was larger when the participants had to perform an overt response task than when they had to imagine the response.

In the analysis of go LRP topography, the ANOVA revealed significant effects for Electrode (*F*_(4,68)_ = 12.2; *p* < 0.001), Task (*F*_(1,17)_ = 9.9; *p* < 0.01), and for the interaction of both factors (*F*_(4,68)_ = 4.8; *p* < 0.01). *Post hoc* comparisons showed that LRP mean amplitude was significantly larger for overt than covert go trials only in fronto-central electrodes (*p* < 0.01 for F3/F4; *p* < 0.001 for FC3/FC4; and *p* < 0.01 for C3/C4) but not in the posterior locations (*p* = 0.081 for CP3/CP4 and *p* = 0.28 for P3/P4). In addition, topographical distribution was similar in both tasks (overt response task: central electrodes > rest of electrode sites except fronto-central electrodes, fronto-central electrodes > frontal and parietal electrodes, and central-parietal electrodes > parietal electrodes; covert response task: fronto-central and central electrodes > central-parietal > frontal and parietal electrodes).

The repeated-measures ANOVA to clarify the effect of successful vs. unsuccessful performance was applied to data from 12 participants, as six of the participants did not produce enough artifact-free US epochs for each hand to yield the LRP. The ANOVA revealed a significant effect of the factor (*F*_(2,22)_ = 6.5; *p* = 0.005), as LRP amplitudes were larger for US than for SS trials (*p* = 0.031) and covert stop trials (*p* = 0.033); however, no differences between the latter two conditions were found (*p* = 1).

The repeated-measures ANOVA (Trial × Task) for LRP onset did not reveal any significant differences for Trial (Fc_(1,17)_ = 0.1; *p* = 0.7), Task (Fc_(1,17)_ = 0.05; *p* = 0.8) or the interaction between these factors (Fc_(1,17)_ < 0.01; *p* = 0.9). The repeated-measures ANOVA with Performance as within-subjects factor did not show a significant effect for LRP onset (*N* = 12) either (Fc_(2,22)_ = 0.03; *p* = 0.9).

### N2 Mean Amplitude

The repeated-measures ANOVA (Trial × Task) did not reveal any significant effect of Trial (*F*_(1,17)_ = 1.1; *p* = 0.3), Task (*F*_(1,17)_ = 0.8; *p* = 0.4) or the interaction between these factors (*F*_(1,17)_ = 0.1; *p* = 0.7).

The repeated-measures ANOVA showed a significant effect of Performance (*F*_(2,34)_ = 10.6; *p* ≤ 0.001). The N2 amplitude was larger for US than for SS trials (*p* = 0.019) and covert stop trials (*p* = 0.002); no differences were found between these two conditions (*p* = 1).

### P3 Mean Amplitude

The repeated-measures ANOVA (Trial × Task) revealed a significant effect of Trials (*F*_(1,17)_ = 11.3; *p* = 0.004). The P3 amplitude was larger in stop than in go trials. The ANOVA did not reveal significant effects of Task (*F*_(1,17)_ = 3.0; *p* = 0.1) nor the interaction between Trial and Task (*F*_(1,17)_ = 3.0; *p* = 0.1).

The repeated-measures ANOVA did not reveal a significant effect of the factor Performance (*F*_(2,34)_ = 1.1; *p* = 0.3).

### Beta ERD (200–550 ms)

**Figure 3:**
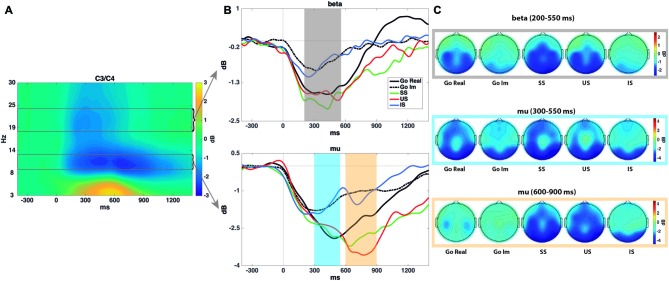
**Time-frequency analyses. (A)** Spectrogram showing the time-frequency power averaged across all conditions in the C3 and C4 electrodes. This plot was used to select time-frequency windows for statistical comparisons. **(B)** Mean mu (9–13 Hz) and beta (18–24 Hz) power for each task and condition–all time-locked to the go signal. As explained in the “Materials and Methods” Section, mu event-related desynchronization (ERD) presents two peaks (especially in stop trials), in both real and imagined performance. Shaded areas encircle the time intervals submitted to statistical analyses. Mu and beta ERD show a similar time course in covert and overt performance, although with a reduced power decrease in the former. **(C)** Topographies of power modulations in each shaded area and condition.

Figure 3 shows the representation of the time-frequency analyses of both beta and mu ERD.

The repeated-measures ANOVA (Trial × Task) revealed a significant effect of Task (*F*_(1,17)_ = 20.6; *p* < 0.001). Beta desynchronization was larger for overt than for covert response trials. The ANOVA did not reveal a significant effect of Trial (*F*_(1,17)_ = 1.3; *p* = 0.3) or the interaction between these factors (*F*_(1,17)_ = 1.9; *p* = 0.2).

The repeated-measures ANOVA revealed a significant effect of Performance (*F*_(2,34)_ = 9.9; *p* < 0.001). A larger decrease in power was found in SS (*p* = 0.001) and US (*p* = 0.021) than in IS trials, but no differences were found between successful and US trials (*p* = 1).

### Mu ERD (300–550 ms)

The repeated-measures ANOVA revealed a significant effect of Task (*F*_(1,17)_ = 7.2; *p* = 0.016). Mu desynchronization was larger in overt than in covert response trials. The ANOVA revealed no significant effect of Trial (*F*_(1,17)_ = 0.1; *p* = 0.8) or the interaction between these factors (*F*_(1,17)_ < 0.001; *p* = 0.9).

The repeated-measures ANOVA revealed a significant effect of Performance on Mu ERD (*F*_(2,34)_ = 4.2; *ɛ* = 0.69; *p* = 0.041), although multiple pairwise comparisons (Bonferroni adjusted) did not reveal any significant differences.

### Mu ERD (600–900 ms)

The repeated-measures ANOVA (Trial × Task) revealed a significant effect of Task (*F*_(1,17)_ = 15.4; *p* = 0.001). Mu desynchronization was larger for overt than for covert response trials. The ANOVA did not reveal a significant effect of Trial (*F*_(1,17)_ = 2.2; *p* = 0.15) or the interaction between these factors (*F*_(1,17)_ = 1.5; *p* = 0.2).

The repeated-measures ANOVA (Performance) revealed a significant effect of the factor (*F*_(2,34)_ = 11.9; *p* < 0.001). A larger decrease in power was observed in SS (*p* = 0.004) and US (*p* = 0.004) than in IS trials, but no differences were found between SS and US trials (*p* = 0.5).

## Discussion

The main goal of the present study was to determine whether a similar pattern of motor-related brain electrical activity is shared in the overt and covert performance of the stop-signal task, a paradigm that exerts strong executive (inhibitory) control. To better capture the power and phase dynamics of the EEG, we included time/frequency analyses (mu and beta ERD) in addition to phase-locked averaged responses (i.e., ERPs).

The results of the present study indicate that covert performance of the stop-signal task appears to recruit neural mechanisms in the brain similar to those used during overt execution and with a similar time course.

The presence of lateralized preparatory activity at central electrodes in the motor imagery condition suggested that M1 is actively involved in the simulated performance of the task. Despite the low spatial resolution of EEG, it is generally considered that the neural source of the LRP component is located at the M1, as revealed by dipole estimation from EEG (Böcker et al., 1994a,b) and MEG studies (Praamstra et al., 1999), and given its inversion of polarity depending on the limb that performs the movement (Brunia, 1980; Carrillo-de-la-Peña et al., 2006). The study findings also confirmed that the temporal pattern of activation is the same in covert and overt performance, as no difference was found in LRP onset between conditions.

It could be questioned whether our LRP results certainly support M1 activation during motor imagery. In fact, it has been argued that, depending on the physical setting of visual stimuli, LRP could reflect lateralized posterior activity rather than motor processing (Praamstra, 2007). In addition, with settings of asymmetric stimuli (as it is the case of arrows), other components related to attentional shifts, as the early directing-attention negativity (EDAN), the anterior directing-attention negativity (ADAN) and the late directing-attention positivity (LDAP; Verleger et al., 2000; Praamstra et al., 2005; Gherri and Eimer, 2010; Praamstra and Kourtis, 2010), or inhibitory mechanisms, as the N2cc component (Oostenveld et al., 2001; Praamstra and Oostenveld, 2003; Praamstra, 2006; Cespón et al., 2012) might also overlap with LRP.

Given that we did not use eccentric settings of stimuli (all were presented in the center of the screen), the contribution of lateralized brain activity associated to stimulus processing might be ruled out. The LRP scalp distribution, with maximal amplitudes between frontocentral and central electrode sites, and reduced amplitude towards more anterior and posterior sites is also inconsistent with reports of the topographical distribution of attention-shifts ERP waves, as EDAN, ADAN and LDAP. In addition, in a previous study using the same array of stimuli (arrows with the same tail and head sizes), we reported an inversion of polarity when the participants performed the task using feet movements (in both overt and covert trials; see Carrillo-de-la-Peña et al., 2006), an effect that supports the contribution of M1 in the generation of LRP (Brunia, 1980; Böcker et al., 1994a,b). In any case, our results support that a similar brain network is involved in real and imagined inhibition, regardless of whether it is referred to M1 activation, activation of frontoparietal networks, or engagement of premotor inhibitory mechanisms.

The amplitude of the LRP was smaller in motor imagery than in the overt motor execution and inhibition, as consistently observed in previous studies (Galdo-Alvarez and Carrillo-de-la-Peña, 2004; Carrillo-de-la-Peña et al., 2006, 2008). Although this might be interpreted as a sign of weaker motor activation in simulated performance, it is open to alternative explanations. As LRP was also smaller in stop trials than in go trials in the overt condition, it could be argued that the smaller LRP amplitudes in motor imagery are due to the presence of larger or sustained motor inhibition during the task. Alternatively, previous studies have also indicated that differences between overt and covert conditions may be due to stimulus processing (Hohlefeld et al., 2011) or the lack of feedback or control from somatosensory areas (Carrillo-de-la-Peña et al., 2008) rather than to motor activation processes.

Results of time-frequency analyses paralleled those found for LRP and provide a complementary view of the temporal dynamics of motor-related EEG in stop-signal tasks. As in previous studies (Pfurtscheller and Neuper, 1997; McFarland et al., 2000), we observed mu and beta ERD over the lateral central electrode sites during motor imagery; again, the decrease in power of those central rhythms was larger in overt performance. Although some studies have related the power of these bands to motor cortex activation, it has also been demonstrated that bilateral mu and beta ERD may be associated specifically with activation of the somatosensory cortex (Jurkiewicz et al., 2006).

In relation to the ERP components characteristic of the stop-signal task, we found that only P3 was significantly larger for stop than go trials, also in the simulated condition. The presence of Stop-P3 in the latter condition suggests that subjects actually canceled an already prepared response even during motor imagery. This result replicates a previous study with auditory stop signals that found similar P3 amplitude and midfrontal theta in imagined than in successfully stopped trials (González-Villar et al., 2016). As explained below, this finding has practical implications and contributes to understand the functional meaning of Stop-P3.

The inhibition of inappropriate responses is an important part of goal-oriented behavior. From a practical point of view, the observed involvement of similar neural circuits in the covert performance of the stop-signal suggests the possibility of training inhibitory skills through mental rehearsal. Non-invasive methods of recording brain signals, such as the EEG, are widely used in BCI. To date, only brain electrical activity indices of motor activation or stimulus detection have been used as BCI communicating systems. Our findings suggest that the indices of inhibition obtained in motor imagery could also be used as communicating systems and could be useful for developing hybrid BCIs that incorporate various sensing modalities in the brain (i.e., detection of directional movement and inhibition of that movement).

Previous studies have found larger N2 and P3 amplitudes for stop than for go trials. These modulations are usually interpreted as reflecting inhibitory control (De Jong et al., 1990; Dimoska et al., 2003, 2006), although it has also been considered that N2 may reflect conflict detection (Carter et al., 1998; Nieuwenhuis et al., 2002, 2003; Donkers and van Boxtel, 2004; Yeung et al., 2004; Enriquez-Geppert et al., 2010), and P3 the evaluation of the inhibitory process, because of its latency (Huster et al., 2013). Nonetheless, other differences between go and stop trials may contribute to the N2 and P3 modulations reported: first, a motor response, including muscular activation, is only present in go trials (and US trials); second, a stop signal is present only in stop trials, and therefore these trials involve double processing (go stimulus and stop stimulus) that may overlap. Thus, the functional significance of Stop-N2 and Stop-P3 is far from clear.

In the present study, two different experimental manipulations were carried out to clarify these alternative explanations: the inclusion of motor imagery to confirm/dismiss the role of motor execution processes (as no overt response is present during the mental essay of the stop-signal task), and the application of a procedure to remove go stimulus-linked activity from stop trials (see “Materials and Methods” Section).

It has been suggested that P3 in no-go trials may be due to the absence of movement-related negativity (Salisbury et al., 2004), and this could be extrapolated to Stop-P3. In the present study, no movement was present in either covert go or stop trials, but a prominent Stop-P3 appeared only in the latter. After comparing a press no-go and a count no-go condition, Smith et al. (2013) also concluded that P3 is due to motor inhibition related positivity in no-go trials. Thus, the presence of Stop-P3 during the imagery condition in the current study ruled out an interpretation based on differences in motor processes. The analysis of stop trials free from the influence of the go signal also allowed us to conclude that the larger amplitude of P3 in stop trials is not due to the summation of activity evoked by two consecutive stimuli.

In the present study, we failed to replicate the larger N2 to stop than to go trials reported in previous studies. However, in a comparison of Stop N2 in successful and US trials, Ramautar et al. (2006) found a larger N2 in unsuccessful trials and indicated that Stop N2 resembled an Error-Related Negativity. Our findings are consistent with this interpretation, as we observed larger N2 amplitude in US trials than in SS trials.

Despite the above contributions, there are some limitations in the experimental design; first, the role of M1 in inhibitory control remains unclear. Further research is required to establish whether M1 acts as a passive receptor of inhibitory signals from other components of the executive control network or assumes an active function in the suppression of motor processing. Since previous studies have considered beta rebound as a correlate of inhibition or return to an idling state after termination of a motor program (Neuper and Pfurtscheller, 1996), even after motor imagery (Pfurtscheller et al., 2005; Solis-Escalante et al., 2012), it would be interesting to analyze beta rebound in stop trials, what requires longer ISIs than the ones used in the present study. Our design was also unable to clarify whether Stop-P3 reflects actual inhibitory control or, alternatively, evaluation of the inhibitory process. As Huster et al. (2013) have argued, this process is initiated and controlled before the culmination of P3, suggesting that the component may reflect evaluation of the inhibitory outcome. Similarly, Wessel and Aron (2015) proposed use of the onset of the frontocentral P3 as a better indicator of response inhibition. Finally, we could not rule out the attentional effect produced by the red arrow (stop) in the N2 and P3 amplitudes. Future studies should include a condition in go trials with a second stimulus as a confirmatory signal (e.g., a green arrow to continue with the motor program).

Overall, the present findings add to previous cumulative evidence for the existence of a shared neural substrate between imagined and executed movements (Stavrinou et al., 2007), supporting the functional equivalence hypothesis (Jeannerod, 2001). The results provide a consistent picture: similar lateralized activity (LRP, mu and beta ERD) was observed both in overt and covert responses, with a similar time course (identical LRP onset, and mu and beta ERD temporal windows) and pattern of task-modulation (differences between go and stop trials). Thus, the results suggest that the mental imagery of a motor plan leads to activation of the same network, with similar temporal dynamics and constraints. The use for the first time of a motor imagery paradigm during performance of a stop-signal task allowed us to further conclude that a similar inhibitory network may be also active during covert execution of the task.

As stated above, this finding could contribute to the development of more sophisticated BCI and provides the scientific basis for understanding the efficacy of motor imagery techniques for improving performance in professional athletes (Jones and Stuth, 1997; Ridderinkhof and Brass, 2015) or motor rehabilitation in patients with neurological lesions (Dickstein and Deutsch, 2007; Zimmermann-Schlatter et al., 2008).

## Author Contributions

SG-A was responsible for the first manuscript draft, manuscript editing and the statistical analyses, and contributed to literature review, and manuscript review. FMB contributed to task design, EEG recording and literature review. AJG-V was responsible for EEG processing and figures, and contributed to literature review and manuscript review. MTC-P was responsible for task design and contributed to statistical analyses, literature and manuscript review. All the authors contributed to interpretation of results.

## Conflict of Interest Statement

The authors declare that the research was conducted in the absence of any commercial or financial relationships that could be construed as a potential conflict of interest.

## Footnotes

^1^The SSRTs represent the point at which the stop process finishes and can be estimated taking into account the go RT distribution and the observed probability of successful/unsuccessful inhibitions to the stop signal for a given SSD (go-stop interval). Using the staircase-tracking algorithm facilitates the estimation of the SSRT since that probability is around 0.50. Thus, it is possible to calculate SSRT by subtracting the observed mean SSD from the observed mean go RT (Logan and Cowan, 1984; Logan et al., 1997).
